# Comparison of two different methods for measuring anti-mullerian hormone in a clinical series

**DOI:** 10.1186/s12958-015-0101-5

**Published:** 2015-09-22

**Authors:** Josephine Hyldgaard, Pinar Bor, Hans Jakob Ingerslev, Niels Tørring

**Affiliations:** Department of Obstetrics and Gynecology, Regional Hospital of Randers, Randers, Denmark; Department of Clinical Biochemistry, Aarhus University Hospital, Skejby Palle Juul-Jensens Boulevard 99, DK-8200 Aarhus, Denmark; The Fertility Clinic, Aarhus University Hospital, Aarhus, Denmark

**Keywords:** Anti mullerian hormone, Assay, ELISA, Variability, CV%

## Abstract

**Background:**

Anti Mullerian hormone (AMH) has previously been measured using a manual method, but a fully automated assay from Roche Diagnostics was recently introduced. The aim of this study was to compare the results from the AMH gen II ELISA and Elecsys Cobas AMH methods in a clinical setting to evaluate whether the assays achieve the goals of analytical performance.

A prospective observational study with 23 women seeking laparoscopic sterilization was conducted. Blood samples were collected preoperatively as well as 1 week and 1, 3 and 6 months postoperatively; they were evaluated with the AMH gen II ELISA and Elecsys Cobas AMH methods. The assays were validated according to the optimal performance of biochemical assays: *CV*_Analytical_ < 0.25* *CV*_Within Biological Variation_.

**Findings:**

We found a good correlation between the two methods; there was a bias of approximately 32 %.

The total within-person biological variability ranged from approximately 21 to 32 %. The analytical variability of the AMH gen II ELISA and Elecsys Cobas methods ranged from 5.5 to 10.3 % and 2.8 to 3.3 %, respectively. Applying the goals for optimal assay performance, the Elecsys Cobas method achieved optimal performance throughout the measuring range, whereas the AMH Gen II only achieved optimal performance in the high end of the measuring range. Furthermore, the Elecsys Cobas assay had a low limit of quantitation of 0.5 pmol/l compared to 3.0 pmol/l for the AMH gen II ELISA.

**Conclusions:**

In the clinical setting, the Elecsys Cobas AMH assay performs well according to the optimal standard for biochemical assays.

## Findings

### Introduction

Anti Müllerian Hormone (AMH) is increasingly being used as a biochemical marker for the assessment of the growing ovarian pool and thus as a surrogate marker for the ovarian reserve and female fertility [1]. AMH was originally introduced as a marker with minimal variation during the menstrual cycle [2, 3] in opposition with other markers that have hitherto been used, such as follicle stimulating hormone (FSH) and inhibin. However, recent publications have demonstrated intra-cycle fluctuations, with the highest levels in the early follicular phase and the lowest levels in the late luteal phase [4], as well as substantial circadian variations [5]. This should be interpreted with caution because the results are based on a small number of samples, and they are influenced by assay performance. It must be considered that the total variation associated with the measurement of AMH is a combination of the within-person variation and a variation linked to the assay itself, which is represented as follows: *CV*_TOTAL_ = √ [(*CV*_Within-person Biological Variation_)^2^ + (*CV*_Analytical_)^2^] [6]. In clinical biochemistry, the setting of analytical goals based on the within-person biological variability is often considered the most appropriate, and the optimal performance (*CV*_Analytical_ < 0.25 *CV*_Within Biological variation_), should be achieved when the technology and methodology are available [6].

Until recently, AMH has been measured with an enzyme-linked immunosorbent assay (ELISA, Beckmann Coulter), which is a manual technique characterized by large analytical variation. However, a new automated assay (Elecsys Cobas, Roche), which shows good intermediate imprecision, has recently been introduced for measuring AMH [7].

The aim of this study was to compare the utility of the two assays in a clinical setting to evaluate whether the assays meet the goals of analytical performance. We investigated the blood samples collected from women who participated in a study examining the potential changes in ovarian reserve following laparoscopic sterilization as an example of minimally invasive pelvic surgery.

## Material and methods

A prospective observational pilot study with a total of 31 women requesting laparoscopic sterilization were included. In total, 23 women with a median age of 36 years (range 33–38 years) completed the study. Patients were included between October 2013 and May 2014. Patients follow-up was conducted until October 2014. Blood samples were analyzed in November/December 2014. The exclusion criteria were endocrine disease, oligomenorrhea (>30 day cycle), polycystic ovarian syndrome (PCOS) (Rotterdam criteria), hormonal contraception, giving birth ≤ 3 months prior to the inclusion date, postmenopausal status, or suspected malignancy.

Blood samples were collected to assess the levels of AMH prior to surgery, one week after surgery and 1, 3 and 6 months after surgery. Serum was isolated within 4 h of sampling and stored at minus 80 °C until analysis.

Ultrasound examination to evaluate both the antral follicle count (AFC) and ovarian volume (OV) was performed.

Laparoscopic sterilization was performed under general anesthesia by a two-port technique. Fallopian tubes were grasped 2 cm lateral to the uterine corners and bipolar coagulation was used.

Ultrasound measurements were performed using the Volusom E6 (6.6 MHz transducer) or the Logiq 9 (8 MHz transducer). The number of follicles that were between 2–10 mm in diameter in both ovaries were determined as well as the average ovarian volume (½*D1*D2*D3).

All biochemical measurements of AMH were performed at the Department of Clinical Biochemistry, Aarhus University Hospital by skilled technicians. The laboratory is licensed according to the ISO 15189 accreditation standard for clinical laboratories.

For the Beckmann Coulter AMH Gen II ELISA, the standard application protocol of employing a pre-mixture of clinical samples, calibrators and controls in assay buffer was used. A standard manual technique was used. The limit of acceptance for daily internal controls in the two levels was CV < 11–14 %. The limit of quantitation (LOQ) (lowest concentration of analyte that can be quantified with a coefficient of variation (CV %) < 15 %) was 3.0 pmol/l. The measuring range without dilution was 3–70 pmol/l. Samples were analyzed in duplicate, and the middle value of two results was used. If duplicate results differed by more than 15 %, the analysis was repeated.

The Elecsys Cobas AMH was analyzed on a Cobas 6000 e601 platform using Roche’s standard protocol. The measuring range without dilution was 0.5–160 pmol/l. The limit of acceptance for daily internal controls in two levels was CV < 5 %, and the LOQ was 0.5 pmol/l.

Spearman’s correlation test was used to determine the correlation between the AMH and total AFC, and the ovarian volume was calculated using STATA 13 software (StataCorp, Texas, USA). Passing-Bablok and Bias plots were made in Analyse-it (Analyse-it Software, Ltd).

The study was approved by the Central Denmark Region Committees on Health Research Ethics, Journal number 1–10–72–260–13, 8th of October 2013.

## Results

Twenty-three of 31 (74 %) women with a median age of 36 years (range 33–38 years) completed the study. The median BMI was 25 (range 22.2–28.7). A total of 112 serum samples from 23 women were included in the study. One woman missed the 3- and 6-month samplings, and one woman missed the 6-month sampling.

### Ovarian reserve

The mean concentrations of AMH measured by Cobas and ELISA correlated significantly with the total AFC and were as follows: at baseline, Cobas AMH vs. AFC: 0.83 (0.6–0.92) (Spearman r (95 % confidence interval)) and ELISA AMH vs. AFC: 0.86 (0.67–0.94); at the 3-month follow-up, Cobas AMH vs. AFC: 0.83 (0.60–0.93) and ELISA AMH vs. AFC: 0.81 (0.56–0.92) (*p* <0.0001). Significant correlations were also observed between AMH measured and the ovarian volume by both Cobas and ELISA (not shown).

AMH, the total AFC, and OV did not change significantly during the 6-month follow-up period from the baseline levels prior to surgery.

### Comparison of the two assays

Analyzing controls on consecutive days, we found a large difference in the analytical variation between the two methods. As seen in Table 1, the analytical variance for the Elecsys Cobas method was approximately 3 % in the high and low ranges compared with the ELISA method, which showed a larger variation in the high range and a particularly larger variation in the low range with a CV of more than 10 %.

**Table 1:** Performance of Beckmann Coulter AMH Gen II ELISA and Elecsys Roche AMH assay

	ELISA (*n* = 38)		Cobas (*n* = 40)	
	Level	CV%	Level	CV%
Control 1	40 pmol/l	5.2	40 pmol/l	3.3
Control 2	20 pmol/l	6.2	-	-
Control 3	7 pmol/l	10.3	7 pmol/l	2.8
Total CV% (<10 pmol/l) (*n* = 11)	31.9 (11.9–47.3)		28.0 (9.5–43.0	
Total CV% (>10 pmol/l) (*n* = 12)	22.0 (11.9–35.2)		21.3 (11.6–33.6)	
Percentage of samples without quantitation	15 %		2 %	

Controls were analyzed on consecutive days of analysis over a period of 3 months. Data are given as CV% (Standard deviation x 100/ mean). Results of variance of clinical samples are given in two levels: < and >10 pmol/l (because of bias between the two methods, the level differ between the two groups.). Data are given as CV% (range). Percentage of samples without quantiation is determined as the percentage of results < 3 pmol/l (ELISA), and < 0.5 pmol/l (Elecsys Cobas)

**Fig. 1:**
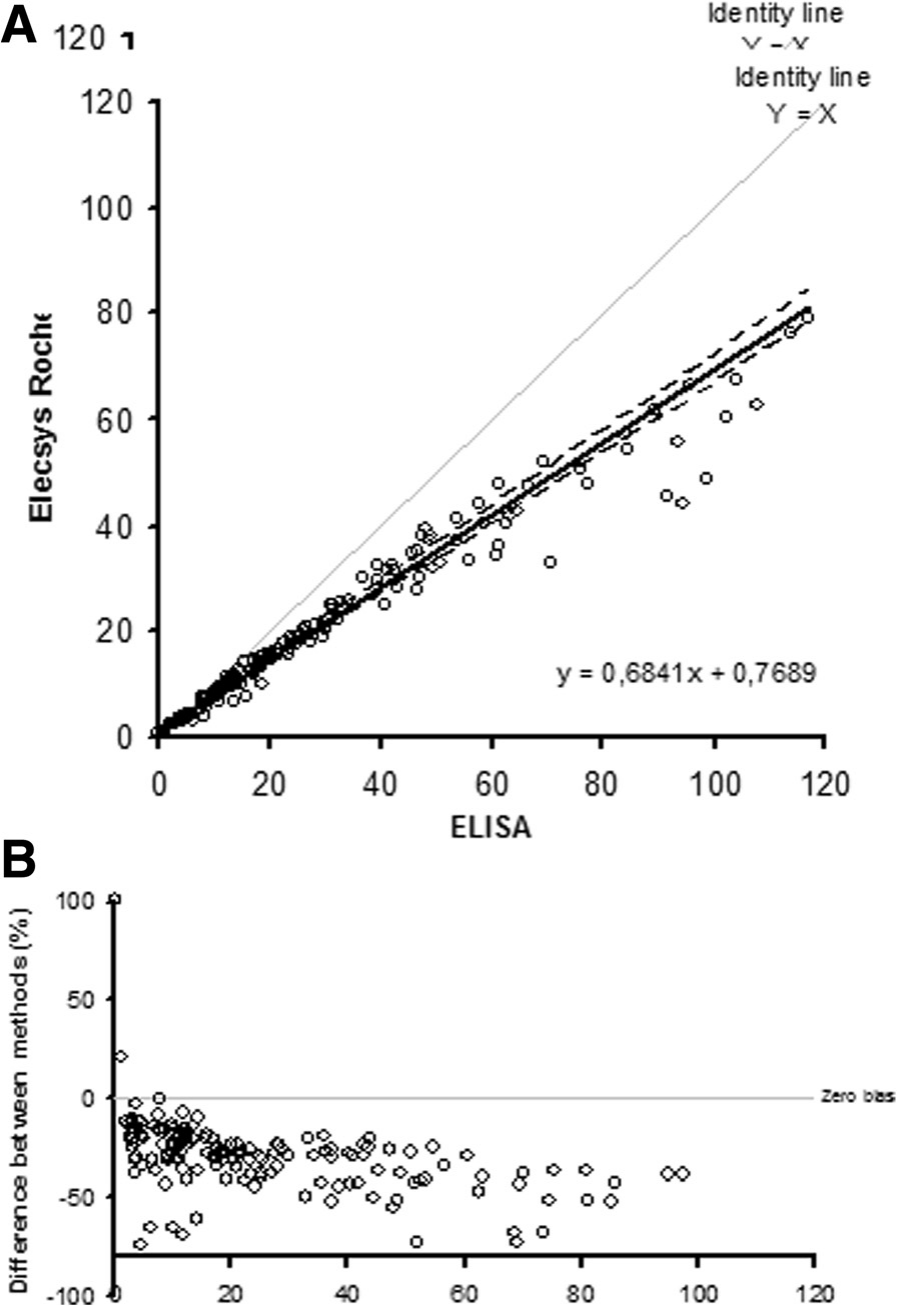
Comparison between the Beckmann Coulter AMH Gen II ELISA and Elecsys Roche AMH (Anti Müllerian Hormone) assays. **a**: Passing-Bablok plot displaying the regression (solid line) and 95 % confidence interval (dotted lines) and **b**: Bias plot displaying the difference between methods (as a percentage). Results are given in pmol/l

As depicted in Fig. 1, the concentrations of AMH measured by the Elecsys Cobas assay were, on average, 32 % lower than the concentrations measured by ELISA (Fig. 1a), and there was a tendency of increased bias between the two assays in the high concentration range (Fig. 1b).

The total variance (biological and analytical) in the clinical samples was divided in two levels, < and > 10 pmol/l. The total variance (mean CV%) in the < 10 pmol/l group was 32 % when measured by ELISA and 28 % when analyzed using the Elecsys Cobas method. For concentrations > 10 pmol/l, the CV was approximately 22 % according to both assays. Applying the goals for analysis (*CV*_Analytical_ < 0.25 *CV*_Within-person Biological Variation_), the analytical variation should not exceed 5.3 % and 7.6 % in the two levels, respectively. This was achieved by the Elecsys Cobas assay throughout the measuring range, whereas the AMH Gen II ELISA failed to meet the goals in the low measuring range.

The limit of quantitation (LOQ) (CV < 15 %) was 3 pmol/l for the ELISA and 0.5 pmol/l for the Elecsys Cobas method. Accordingly, approximately 15 % of the measurements were below the LOQ for the ELISA assay, but only 2 % were below the LOQ for the Elecsys Cobas method.

## Discussion

The main purpose of the present report was to compare two assays for measuring AMH in a clinical setting.

As expected, there were no changes in the ovarian reserve parameters (AMH, AFC or OV) during a follow-up period of 6 months. Comparing the AMH Gen II ELISA and Elecsys Cobas methods, we found an almost constant bias between the two assays. Judging from the external quality assessment program for AMH (UKNEQAS peptide hormones (WWW.UKNEQAS.ORG.UK)), the bias between the two assays is in the range of 5–15 %. Our laboratory has previously observed a positive bias using the same program of approximately 20 % when we reported results for the AMH Gen II ELISA assay. Therefore, interlaboratory bias may account for the observed bias, which is somewhat larger than previously published [7]. We found a total variation in the repeated analysis over 6 months ranging between 21 % and 32 % depending on the level of the measurements and the assay. This means that the within-person biological variation ranges between 21 % and 30 %. This variation is similar to data from Rustamov et al., who reported a 28 % within-person biological variation in a population of 186 subfertile patients with repeated measurements of AMH (median interval of 2.6 months between samples) [8].

Because the Elecsys Cobas assay can reliably measure concentrations as low as 0.5 pmol/l, it is now possible to achieve precise quantitative measurements for a low level of AMH (<3 pmol/l). This can be beneficial and useful for clinical evaluation of the ovarian reserve of women before undergoing assisted reproductive technology or in the case of imminent menopause.

## Conclusion

There was a good correlation between the Elecsys Cobas AMH and AMH Gen II ELISA methods for the entire measuring range. While the goals for optimal analytical performance were achieved with the Elecsys Cobas assay, they were not met with the AMH Gen II ELISA assay. This is important in the clinical setting because women seeking treatment for infertility often have a low AMH; therefore, analysis with Elecsys Cobas will provide a better option for assessing AMH in this group of women in addition to its ability to measure levels as low as 0.5 pmol/l.

## Abbreviations

AMH: Anti müllerian hormone
FSH: Follicle stimulating hormone
AFC: Antral follicle count
OV: Ovarian volume
CV: Coefficient of variation

## References

[1] La Marca A, Broekmans FJ, Volpe A, Fauser BC, Macklon NS (2009). ESHRE Special Interest Group for Reproductive Endocrinology--AMH Round Table. Anti-Mullerian hormone (AMH): what do we still need to know?. Hum Reprod.

[2] Hehenkamp WJ, Looman CW, Themmen AP, de Jong FH, Te Velde ER, Broekmans FJ (2006). Anti-Mullerian hormone levels in the spontaneous menstrual cycle do not show substantial fluctuation. J Clin Endocrinol Metab.

[3] Visser JA, de Jong FH, Laven JS, Themmen AP (2006). Anti-Mullerian hormone: a new marker for ovarian function. Reproduction.

[4] Hadlow N, Longhurst K, McClements A, Natalwala J, Brown SJ, Matson PL (2013). Variation in antimullerian hormone concentration during the menstrual cycle may change the clinical classification of the ovarian response. Fertil Steril.

[5] Bungum L, Jacobsson AK, Rosen F, Becker C, Yding Andersen C, Guner N (2011). Circadian variation in concentration of anti-Mullerian hormone in regularly menstruating females: relation to age, gonadotrophin and sex steroid levels. Hum Reprod.

[6] Fraser CG, Hyltoft Petersen P, Libeer JC, Ricos C (1997). Proposals for setting generally applicable quality goals solely based on biology. Ann Clin Biochem.

[7] Gassner D, Jung R (2014). First fully automated immunoassay for anti-Mullerian hormone. Clin Chem Lab Med.

[8] Rustamov O, Pemberton PW, Roberts SA, Smith A, Yates AP, Patchava SD (2011). The reproducibility of serum anti-Müllerian hormone in subfertile women: within and between patient variability. Fertil Steril.

